# A method for supporting retrieval of articles on protein structure analysis considering users’ intention

**DOI:** 10.1186/1471-2105-12-S1-S42

**Published:** 2011-02-15

**Authors:** Riku Kyogoku, Ryo Fujimoto, Tomonobu Ozaki, Takenao Ohkawa

**Affiliations:** 1 Graduate School of System Informatics, Kobe University, Rokkodai, Nada, Kobe 657–8501, Japan; 2 Graduate School of Engineering, Kobe University, Rokkodai, Nada, Kobe 657–8501, Japan; 3 Cybermedia Center, Osaka University, Yamadaoka, Suita 565–0871, Japan

## Abstract

**Background:**

In recent years, information about protein structure and function is described in a large amount of articles. However, a naive full-text search by specific keywords often fails to find desired articles, because the articles involve the ambiguous and complicated concepts that cannot be described with uniform representation. For retrieving articles on protein structure and function, it is important to consider the relevance between structural and/or functional concepts by identifying the user’s intention.

**Results:**

We introduce a scheme of evaluating relevance between articles based on various biological databases and ontologies on structures and functions of proteins. The relevance, which is defined as a path length between concepts on hierarchies, is modified adaptively based on additional articles as a query in order to reflect the user’s intention. Also we implemented the retrieval system, in which the user can input some articles as a query and the related articles are retrieved and displayed on the 2D map.

**Conclusions:**

The effectiveness of the proposed system was confirmed experimentally by having shown that the users can obtain easily highly related articles which reflect their intention.

## Background

In recent years, information about protein structure and function is described in a large amount of articles by the progress of the study on the protein structure analysis. In order to use these huge amount of articles as information repository, the system in which the articles are collected and arranged suitable for retrieval is required. However, a naive full-text search by specific keywords often fails to find desired articles, because the articles involve the ambiguous and complicated concepts that cannot be described with uniform representation. For retrieving articles on protein structure and function, it is important to consider the relevance between structural and/or functional concepts. In addition, since an article can be treated from various aspects (e.g. methodology, target protein, disease, etc.), it is also important to identify the user’s intention for the retrieval requirement in order to find out appropriate articles based on the input articles as a query.

In this paper, we propose a new method of supporting the retrieval of the related articles taking into account the user’s intention. In our method, one or more articles are treated as an input query, and the relevance between articles are evaluated from various viewpoints such as protein structure and function involved in the articles. The relevance between articles is calculated by using their concept hierarchy.

Especially, in order to clarify user’s individual intentions of retrieval, the value of relevance is modified adaptively using more than one articles (an initial article and additional articles) as an input query. That is, the relevance between any two concepts that are linked through the path between the concepts described in the initial article and the additional articles is updated to be regarded as more similar.

In addition, we have implemented the method mentioned above to develop a system for retrieving related articles from the dynamically identified user’s intention. In this system, the user can input some articles as a query, and the related articles are retrieved and displayed on the 2D map to understand the relation between retrieved articles visually.

There are many methods for retrieving the similar articles, such as Google Scholar [1], CiteSeer [2] and GoPubMed [3], etc. Google Scholar is a website which can retrieve the academic articles easily, and can show the most related articles in the index. The related articles are sequentially displayed from the article that has many features shared with the key article, and the mutual relativity between the articles is also considered. CiteSeer is a system which retrieves the information of the reference from academic articles, and can extract the co-citation relation between the articles [4]. GoPubMed is a retrieval method based on the relevance between the keyword and the GO, and it can make the results which are easy to understand for users because of the categorizing by GO [5].

In such systems, although keywords are the primary input as a query, the related articles can also be retrieved based on the retrieved article. However, these systems cannot understand the user’s intention that should be considered for the effective retrieval. In our method, the relevance between articles is evaluated based on multiple aspects, and the user’s intention is dynamically specified by extracting the related features between input articles given as a query.

## Methods

In this paper, we deal with retrieval of the articles referred from each entry of the PDB (Protein Data Bank), because we will use the structural information related to the article. If you need not attach great importance to structural aspect, this restriction is not always required. For each article, based on the corresponding PDB entry, the structural and functional information on the objects (e.g. protein, gene), concepts, themes, and so on described in the article is referred from various bio-databases as follows,

• A database of protein structure information (PDB)

• A database of protein structural classification information (SCOP)

• A database of gene ontology (GO)

• A database of protein sequence information (Swiss-Prot/Uni-Prot)

• A database of biomedical article (MED-LINE/PubMed)

The related articles can be retrieved based on the relevance between input articles and the target articles, which is evaluated using the information and conceptual hierarchies obtained from these databases.

### Calculation of the relevance based on the concept hierarchy

#### Evaluation of relevance using the concept hierarchy

Before defining the relevance between articles, we define *d*(*H*, *t*_1_, *t*_2_), the relevance between two concepts *t*_1_ and *t*_2_ on a concept hierarchy *H* as(1)

where *d*(*H*) represents the depth of the concept hierarchy and *c_p_*(*t*_1_, *t*_2_) represents the lowest level ancestor common to *t*_1_ and *t*_2_. *P*(*t*, *c_p_*(*t*_1_, *t*_2_)) is the path length between *t* ∈ *t*_1_, *t*_2_ and *c_p_*(*t*_1_, *t*_2_) defined as follows.

*P*(*t*, *c_p_*(*t*_1_, *t*_2_)) = min{|*E*| | *E* ∈ *P*_min_(*t*, *c_p_*(*t*_1_, *t*_2_))} (2)

where *P*_min_(*t*, *c_p_*(*t*_1_, *t*_2_)) is the set of the shortest paths from *t* to *c_p_*(*t*_1_, *t*_2_). The length of each edge is assumed to be a fixed value, namely 1.0, to make understanding easier, but we redefine the equation (2) by giving weight to each edge in order to reflect the user’s intention to the relevance evaluation process. The redefinition by giving weight to update the edge length is described later.

**Figure 1:**
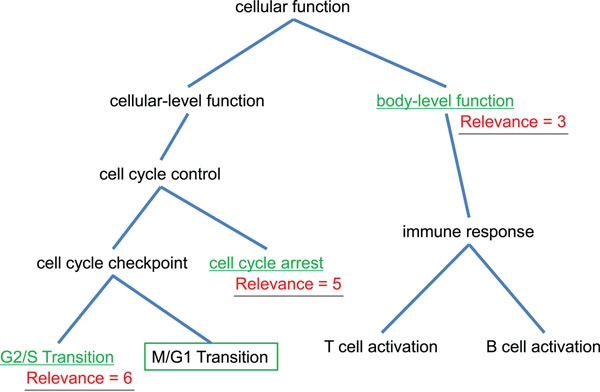
**An example of the evaluation of the relevance between concepts**. The relevance between “M/G1 Transition” and “cell cycle arrest” is 5 (= 4 × 2 – 3) because the depth of concept hierarchy is 4 and the sum of path to the lowest common ancestor “cell cycle control” is 2+1.

Figure 1 shows an example of relevance evaluation by the concept hierarchy. For example, the relevance between two concepts “M/G1 Transition” and “cell cycle arrest” is 5 (= 4 × 2 – 3) because the depth of concept hierarchy is 4 and the sum of path to the lowest common ancestor “cell cycle control” is 2+1. The relevance evaluation between the articles on the concept hierarchy of GO (Gene Ontology) and SCOP based on the equation (1) is discussed in succeeding sections.

#### Calculating the relevance between concepts from the functional viewpoint

The relevance between concepts in GO is evaluated based on the idea that “the gene product related to the lower concept has to be related to the higher concept”. First, the functional concept included in the article is identified by finding the functional information such as keywords or protein names from the GO hierarchy [6,7].

**Figure 2:**
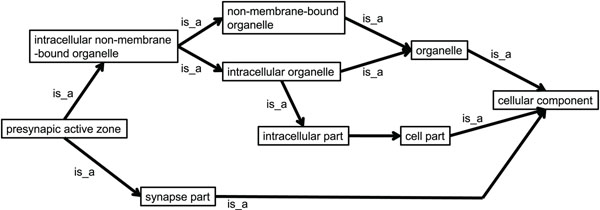
**Concept hierarchy extracted by tracking back “is_a” relations.** A part of hierarchy can be extracted by tracking back the relation “is_a” from a term corresponding to a concept in the article to top of the hierarchy.

Two types of relation, namely “is_a” and “part_of” are used together in one concept hierarchy in GO. Because the relation “part_of” has the ambiguity, only the relation “is_a” is considered for the calculation of relevance in the concept hierarchy. Figure 2 shows an example of hierarchy extracted by tracking back the relation “is_a” based on the concept hierarchy of GO from a term corresponding to a concept in the article to the top of the hierarchy.

If more than one functional concepts (functional terms) are included in one input article, the retrieval results may change depending on which concept is important for the user. Therefore, we introduce the weight that should be assigned to the concept, which is given by the user. We define *d_GO_*(*H*, *D*_1_, *D*_2_), the relevance between articles *D*_1_(input) and *D*_2_(retrieval target) for the functional concept hierarchy in GO as follows.(3)

where *ω*_*t*_1__ is the weight that is assigned to the concept (functional term) *t*_1_ in the article *D*_1_ given by the user, and *GO*(*D*) is the set of the functional concepts (functional terms) in the article *D.*

#### Calculating the relevance from the viewpoint of the protein structure

The protein structures are classified at six levels (i.e. class, fold, superfamily, family, protein, and species) in the SCOP database hierarchically. *d_SCOP_*(*H*, *D*_1_, *D*_2_), the relevance between two articles *D*_1_ (input) and *D*_2_ (retrieval target) based on the protein structural concept hierarchy *H* is defined as(4)

where *SCOP*(*D*_1_) is the set of the structural concepts, namely the classification results of the protein described in the article *D*_1_.

#### Calculating the relevance from the viewpoint of the medical term

The hierarchy consisting of the medical terms can be obtained from PubMed. The MeSH (Medical Subject Headings) terms corresponding to the article are extracted by referring PubMed. MeSH is the National Library of Medicine’s comprehensive controlled vocabulary thesaurus, in which the descriptors are arranged in a hierarchy. Because about ten MeSH terms are related to each article, the same manner in case of GO can be applied for calculating the relevance. In other words, the calculated path length on the hierarchy of the MeSH term for each related articles is used for evaluating the relevance in the input article. And we define the relevance for the viewpoint of the medical term between the articles as the sum of the relevance calculated from each MeSH term.

*d_mh_*(*H*, *D*_1_, *D*_2_), the relevance between two articles *D*_1_ and *D*_2_ based on the protein functional concept hierarchy *H* is defined as(5)

where *ω*_*t*_1__ is the weight of the concept added if the term *t*_1_ is a Major Topic in the article *D*_1_.

### Calculation of the relevance for the related article by the update of the edge length

#### Outline

Generally, in information retrieval system, multiple keywords (namely, AND-search) are often used to specify the user’s requirement. In the proposed method, the user’s intention for retrieval of related articles is specified using more than one articles as inputs (an initial article and additional articles). In other words, the length of the path between concepts, which is calculated using the initial article first, is updated based on the similarity between initial article and additional articles. Note that we use the term ‘relevance’ for evaluating the relation between a concept in query articles and a concept in retrieval targets, whereas we use the term ‘similarity’ for evaluating the relation between concepts in query articles (the initial article and the additional articles) to distinguish them.

#### Calculating the similarity between concepts in query articles

For the concept hierarchy, the attempt to evaluate the similarity between concepts has been well studied. One of the most primary method is to evaluate the length of the path on the graph representing the concept hierarchy with nodes and edges, which is similar manner to our relevance measure mentioned above. But it is difficult to give a weight to the path systematically from only the path length between the concepts. In addition, such a method cannot consider the depth of the common ancestor of two concepts in hierarchy, that is, the similarity is evaluated independently whether the common ancestor is located near the root or remote from the root. Similarly, the method in which the similarity is evaluated based on the entropy focusing on the common ancestor of the concepts has been proposed [8,9]. This method can calculate the similarity considering the location of the common ancestor, but it is not enough to give weight to the path between the concepts which is required for updating the relevance in our method. Therefore, in this study, the method proposed in [10] that can measure the similarity between the concepts considering both the path between the concepts and the common node is applied to weighting the path between the concepts.

#### Giving the weight to the edge in the concept hierarchy

The user’s intention, from which the related articles are retrieved, should be specified by the initial article and the additional articles. By considering the path between the concepts described in the initial article and the additional articles, the article including a lot of concepts similar to the common concepts in them has to be more related to the input articles. Therefore, the weight of the edges connecting the concepts in the initial and the additional articles is given by using the similarity measure described in [10].

We define the weight *ω*(*e*) of the edge *e*, called *edge weight*, using the concept hierarchy as(6)

where *t*_1_ is the concept in *T_A_*, the set of concepts in the initial article *A* and *t*_2_ is the concept in *T_L_*, the set of concepts in the additional article *L. S*(*t*_1_, *t*_2_) is similarity between two concepts *t*_1_ and *t*_2_ calculated by using the method described in [10], and *E* (*t*_1_, *t*_2_) is the set of edges connecting the concepts *t*_1_ and *t*_2_.

**Figure 3:**
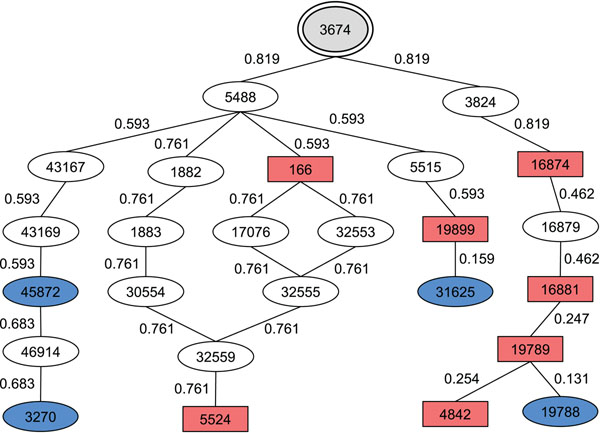
**Edge weights using the similarity**. The edge weight is assigned to each edge in the paths between the concepts. The nodes in red show the concepts in the initial article, and the nodes in blue show the concepts in the additional article.

Figure 3 shows an example of calculated edge weight that is assigned to each edge in the paths between the concepts. The nodes in red show the concepts in the initial article, and the nodes in blue show the concepts in the additional article.

#### Updating edge weight by more than one additional articles

AND-search or OR-search by using more than one additional articles updates the edge weight by using the concept involved in each additional article.

Let *W E*(*A*, *L*) be a set of edges, which the edge weight should be assigned to by the input article *A* and the additional article *L*, as follows.(7)

If the additional articles *L*_1_,…,*L_m_* are given instead of one additional article *L*, only the common edge  is the edge which the edge weight is assigned to in case of the AND-search. If the different edge weight is assigned to the same edge, the smallest one is selected.

On the other hand, the edge  is the edge which the edge weight is assigned to in case of the OR-search. And in the same way as the AND-search, the smallest edge weight is employed in case of adding the different edge weight to the same edge.

**Figure 4:**
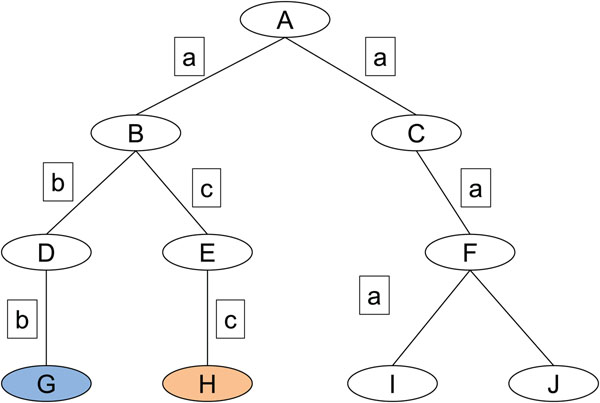
**Selection of weighted edge by the AND-search**. The concept “I” is the concept in the initial article and the concepts “G” and “H” are the concepts in the additional articles. The edge weight is assigned to each of the common edge (denoted by  ) for the AND-search.

In Figure 4, the concept “I” is the concept in the initial article and the concepts “G” and “H” are the concepts in the additional articles. The edge weight is assigned to each of the common edge (denoted by  in the figure) in the intersection of the set of the edges connecting ‘I’ and ‘G’ and the set of the edges connecting ‘I’ and ‘H’ for the AND-search.

#### Evaluating the relevance between two concepts based on updated edge weight

We have defined the calculation of the basic relevance as the equation (1) introduced in the previous section. By adding the edge weight to the path between the concepts based on the additional articles, the definition of *P*(*t*_1_, *c_p_*(*t*_1_, *t*_2_)) and *P*(*t*_2_, *c_p_*(*t*_1_, *t*_2_)) in the equation (1) has to be modified. That is, the equation (2) is redefined as

where *ω*(*e*) is the edge weight from the equation (6) and *P*_min_(*t*, *c_p_*(*t*_1_, *t*_2_)) is the set of the shortest paths connecting *t* and *c_p_*(*t*_1_, *t*_2_).

The equation (2’) is applied to each calculation of the relevance between concepts. In other words, the relevance of the equation (1) is modified by the equation (2’), consequently the edge weight reflecting the user’s intention for the equations (3), (4), (5) is applied.

## Implementation

**Figure 5:**
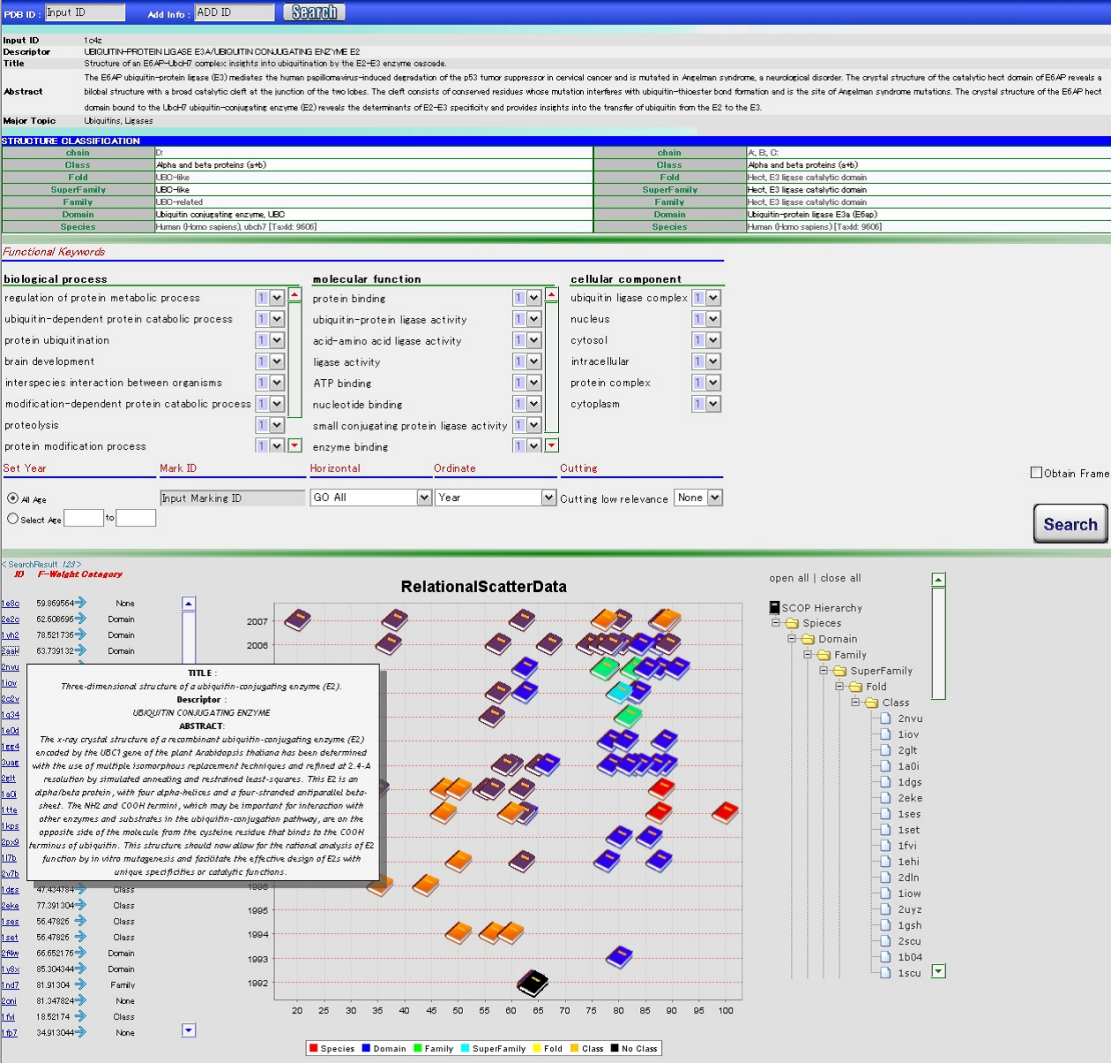
**User interface of the proposed system**. The user can input some articles as a query by using PDB_IDs and the related articles are retrieved and displayed on the 2D map.

Figure 5 shows the display snapshot of the proposed system. In this system, to consider structural information as well as functional information of proteins, the retrieval targets are limited to the articles discussing protein structures. Therefore, only the article registered in PDB is treated as an input. However, because the retrieval algorithm does not depend on the structural information, the core method in the system can be easily extended so as to treat other proteins whose structures have not been analyzed yet. In the current version of the system, for the sake of convenience (and for the sake of evaluation of the proposed algorithm), we give the PDB_ID to identify an input article. In the succeeding system, we plan to extend the target articles and introduce a new framework for inputting queries.

The input of PDB_ID provides the information of the article such as the bibliographic information, the classification of the protein structure from SCOP, the functional information from GO, and MeSH term and the summary from PubMed, and shows them on the browser. Each concept in the initial article can be weighted by user’s manual weight assignment to focus on his explicit interest. In addition, additionally input articles help understand the user’s implicit interest, namely the user’s intention. Evaluating the relevance based on the updated edge weight quantifies the relation to other article and shows the related articles on a map.

## Results and discussion

**Figure 6:**
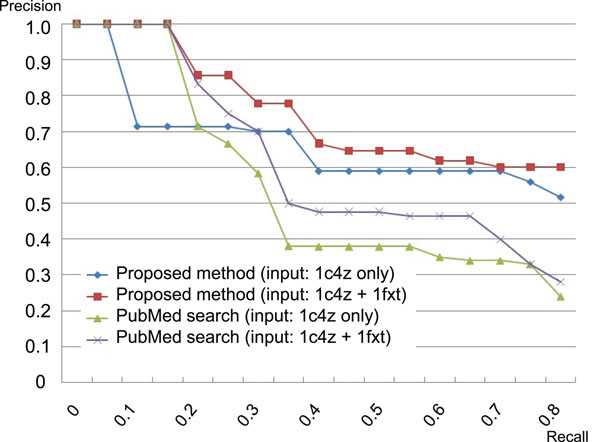
**Precision-recall curve of the search result.** The precision-recall curves are plotted from the retrieval results for two articles “1c4z” and “1fxt” in combination of two types of the methods (proposed method vs PubMed search) and two types of the queries (input article only vs input and additional articles).

For evaluation of the proposed method, we compare the retrieval results by the proposed system with the PubMed search, very popular retrieval system for biomedical articles. In this experiment, the correct article is defined as the article which is cited by one or more articles co-citing two given articles that play a role of the query. Figure 6 illustrates the precision-recall curve [11] arranged from the retrieval result for two articles “1c4z” (initial) and “1fxt” (additional) as one of the example of the experimental results.

This graph shows that the proposed method using the additional query article gives very good result in comparison with PubMed search. The related articles can be detected before giving the additional article, but the correct articles are not really retrieved on high ranking. Adding the new article improves drastically the accuracy of the retrieval.

We evaluate the retrieval accuracy for the combination of other query articles by using average precision (AP). Table 1 summarize the results. The proposed method gives better results than the PubMed search for most input pairs. The MAP (Mean Average Precision) values, which can be calculated from the AP values in Table 1, are 0.725 and 0.660 for the proposed method and the PubMed search respectively. These results show that updating the relevance by adding new articles achieves retrieval of the related articles reflecting user’s intention, which suggests the effectiveness of the proposed method.

**Table 1:** Summary of the retrieval accuracy for proposed method and PubMed search

Query articles		Average precision		Query articles		Average precision	
Initial	Added	Proposed	PubMed	Initial	Added	Proposed	PubMed

1c4z	1fxt	**0.76**	0.64	1fxt	1j7d	**0.72**	0.57
1c4z	1fbv	**0.78**	0.68	1fxt	1vcb	**0.83**	0.74
1c4z	1ayz	0.68	**0.73**	1fxt	1s1q	**0.58**	0.53
1c4z	1y8q	**0.78**	0.53	1fxt	1euv	**0.63**	0.51
1c4z	1nd7	**0.78**	0.53	1fxt	1fqv	**0.79**	0.78
1c4z	1u9a	0.69	**0.82**	1ldk	1p22	**1.00**	0.91
1c4z	1kps	**0.77**	0.60	1ldk	2hye	0.77	**0.81**
2uyz	1tgz	**0.68**	0.55	1ldk	1lm8	0.73	**0.82**
2uyz	2iy0	**0.69**	0.60	1ldk	1r4m	0.60	**0.78**
2uyz	1y8r	**0.81**	0.65	1ldk	1z5s	**0.61**	0.50
2uyz	2nvu	**0.65**	0.57	1yov	1tt5	0.68	**0.84**
2uyz	2eke	0.66	**0.92**	1yov	1jw9	**0.80**	0.68
2uyz	1wyw	**0.76**	0.58	1yov	1mn3	**0.65**	0.46
2uyz	2asq	**0.72**	0.52	1yov	1fqv	0.63	**0.65**
1fxt	1c4z	**0.76**	0.64	1yov	2px9	**0.78**	0.68

## Conclusions

In this paper, a new method for retrieving related articles from multiple query articles and the implemented retrieval system have been presented. In our system, the user can easily retrieve the articles based on the user’s individual intention. In addition, the system can display the retrieval results on the 2D map and it is easy to catch that relation from various viewpoints.

Because the evaluation approach is not enough, one of our future work is making a corpus manually, and then we will evaluate the accuracy using the corpus through the comparison with other methods. Another future work is to target the article which is not referred from PDB, so we will introduce the techniques to extract the sentences describing the functional information from the articles automatically [12,13].

## Competing interests

The authors declare that they have no competing interests.

## Authors' contributions

RK carried out the arrangement of the data set and the experimental results and drafted the manuscript. RF carried out the implementation of the algorithm and performed experiment. TO participated in the algorithm development and the design of the study. TO conceived of the study, and participated in its design and coordination and helped to draft the manuscript. All authors read and approved the final manuscript.
